# Novel technique of switching TIVA and sevoflurane during epilepsy surgery for combined intraoperative motor evoked potentials monitoring and electrocorticography: an illustrative case report

**DOI:** 10.1186/s40981-024-00740-1

**Published:** 2024-09-19

**Authors:** Yoko Mukoyama, Junko Ichikawa, Makiko Komori, Mitsuharu Kodaka, Suguru Yokosako, Yuichi Kubota

**Affiliations:** 1 Departments of Anesthesiology, Tokyo Women’s Medical University Adachi Medical Center, 4-33-1 Kouhoku, Adachi-ku, Tokyo 123-8558 Japan; 2 Department of Neurosurgery, Tokyo Women’s Medical University Adachi Medical Center, 4-33-1 Kouhoku, Adachi-ku, Tokyo 123-8558 Japan

**Keywords:** Epilepsy surgery, Neuroanesthesia, Propofol, Sevoflurane, Anesthetic agent switching, Intraoperative neurophysiological monitoring, Motor evoked potentials, Electrocorticography

## Abstract

**Background:**

During epilepsy surgery, it is equally important to record electrocorticography (ECoG) for detecting epileptogenic activity and guiding brain resection, and to evaluate neuromonitoring data, particularly motor evoked potentials (MEP), for avoidance of postoperative neurological complications. However, sevoflurane, which is commonly used during recording of ECoG, may attenuate the MEP response. It enforces anesthesiologists and neurosurgeons to select one anesthetic agent over another, facilitating either ECoG or MEP monitoring.

**Case presentation:**

In the presented case of a 20-year-old man, who underwent surgery for temporal lobe epilepsy, a novel technique of neuroanesthesia was introduced, integrating initial induction of the total intravenous anesthesia (TIVA) with propofol (effect-site concentration, 2.3–3.0 μg/ml), its subsequent switching to sevoflurane (end-tidal concentration, 2.5%) for ECoG recording, and further change back to TIVA for MEP monitoring during brain resection.

**Conclusions:**

Intraoperative switch of anesthetic agents according to specific intraoperative requirements may be useful for cases of brain surgery requiring both ECoG recordings and MEP monitoring.

## Background

Pharmacoresistant epilepsy accounts for approximately 20–30% of all epilepsy cases and necessitates consideration of the surgical intervention as a possible treatment option, which may be directed at resection or disconnection of the epileptogenic brain, or at neuromodulation of specific neuronal structures [1]. Resective epilepsy surgery requires accurate localization of the epileptogenic zone, which is achieved preoperatively with invasive chronic electroencephalography (EEG) monitoring by means of implanted subdural grid/strip electrodes and/or stereo-EEG (SEEG) with implantation of stereotactic depth electrodes into neuroanatomical structures of interest. Additionally, intraoperative electrocorticography (ECoG) is frequently utilized for detecting epileptogenic activity, guiding the extent of brain resection, and controlling of the epileptic focus elimination.

It is well recognized that an increased concentration of sevoflurane induces epileptic spikes, which facilitates ECoG recording [2]. On the other hand, inhalational anesthetics, such as sevoflurane or desflurane, suppress amplitude of motor evoked potentials (MEP), which constitute a key modality of the contemporary intraoperative neurophysiological monitoring directed at avoidance of postoperative neurological complications after surgical procedures performed under total intravenous anesthesia (TIVA) with propofol and remifentanil. It creates a dilemma and enforces anesthesiologists and neurosurgeons to make an uneasy choice in selecting one anesthetic agent over another, facilitating either ECoG or MEP monitoring. To avoid such a problem, we have introduced a novel technique of switching TIVA and sevoflurane during resective epilepsy surgery for combined intraoperative MEP monitoring and ECoG recording. This experience is highlighted in the presented illustrative case report.

## Case presentation

A 20-year-old man (body height 180 cm, weight 80 kg) was admitted for surgical treatment of pharmacoresistant epilepsy presented with generalized seizures and mental retardation. Previous treatment with a variety of antiseizure medications, including levetiracetam, sodium valproate, clonazepam, and perampanel, was ineffective. Clinical examination, including video-EEG and SEEG monitoring, revealed that seizures originated from the right frontal and temporal lobes, including mesial temporal lobe structures (amygdala and hippocampus). Thus, resective epilepsy surgery guided by ECoG under general anesthesia was planned. Although awake craniotomy with intraoperative cortical mapping has been effectively applied for elimination of the epileptic focus located within or in close vicinity to the eloquent cortex, mainly in cases of lesional pathology [3, 4], it was not considered suitable in our patient, neither was feasible because of his psychiatric disorder. On the day of surgery, without premedication anesthesia was induced with fentanyl 100 μg, target-controlled infusion (TCI) of propofol 3.0 μg/ml, remifentanil 0.25 μg/kg/min, and rocuronium 50 mg. After intubation, sugammadex 200 mg was given to partially antagonize muscle relaxant effects. Starting from the skin incision and until the opening of the dura mater, TIVA was provided by TCI of propofol 2.5 μg/ml and remifentanil 0.25 μg/kg/min, and continuous infusion of remifentanil (0.25 μg/kg/min), which were attained with the syringe pump systems (TE-372; Terumo Co., Tokyo, Japan) based on the Marsh pharmacokinetic model and Minto pharmacokinetic model, respectively. Exact blood concentrations of propofol during surgery were not evaluated, but its dynamic effect-site concentrations were estimated using simulation software TIVA Trainer™ (AQAI GmbH, Mainz, Germany). During surgery, phenylephrine was administered continuously with a target mean arterial pressure of 55–65 mmHg. Respirator parameters included tidal volume of 600–650 ml and breath rate of 12–14 per minute, with a target to maintain PetCO_2_ of 30–32 mmHg. MEP were monitored from the thenar and anterior tibial muscles of the left extremities by means of a Neuromaster neurophysiological monitoring system (MEE-1000; Nihon Kohden, Tokyo, Japan).

**Fig. 1:**
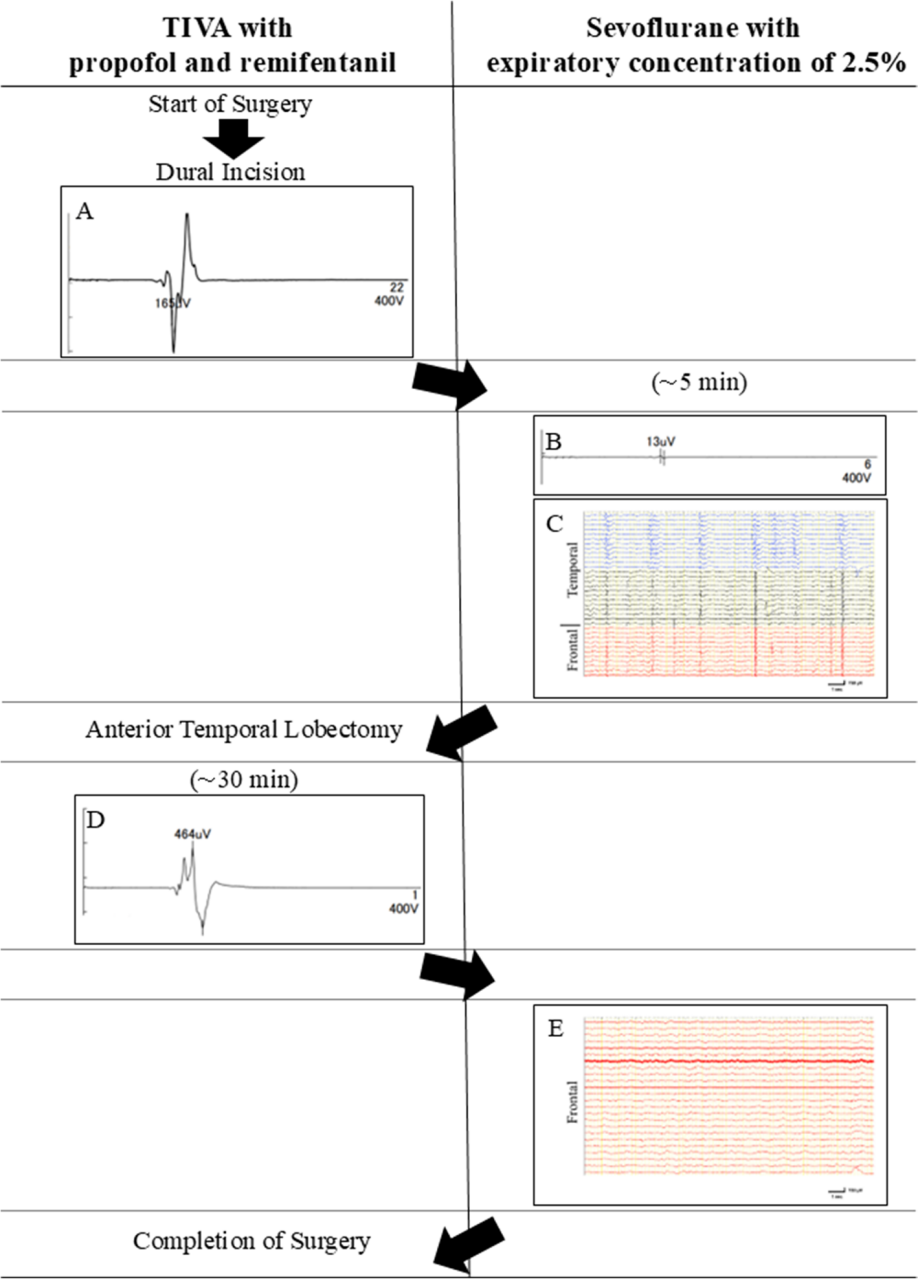
The scheme of anesthesia workflow in the presented case of resective surgery for pharmacoresistant epilepsy characterized by generalized seizures and mental retardation. Starting from the skin incision and until opening of the dura mater, total intravenous anesthesia (TIVA) was provided by means of target-controlled infusions of propofol and remifentanil. The baseline motor evoked potentials (MEP) responses were assessed after dural incision (**A**). It was followed by switching of anesthesia to sevoflurane with its end-tidal concentration of 2.5%. Approximately 5 min later, MEP response had practically disappeared (**B**) and electrocorticography (ECoG) demonstrated frequent synchronized high-amplitude repetitive spikes (**C**) recorded from nearly all leads of the used grid electrode covering both frontal (premotor and motor cortex) and temporal (anterior two-thirds of the temporal lobe) convexity. Within approximately 30 min after return to TIVA, MEP amplitude restored prominently, and did not demonstrate changes of amplitude or latency during subsequent anterior temporal lobectomy (**D**). Thereafter, propofol administration was interrupted again, the end-tidal sevoflurane concentration was changed to 2.5%, and control ECoG showed complete cessation of the previously observed epileptogenic activity from the frontal cortex (**E**), which allowed to avert additional brain resection

The baseline MEP was assessed after a dural incision (Fig. 1A) under TIVA. Thereafter, propofol administration was interrupted and end-tidal sevoflurane concentration was set at 2.5% (Table 1). Short-time decrease of bispectral index (BIS) value from 43 to 34 was noted, but it was restored quickly. Five minutes later, a prominent decrease and, subsequently, complete disappearance of MEP responses from both the upper and lower extremities was noted (Fig. 1B). ECoG showed frequent synchronized high-amplitude repetitive spikes recorded from nearly all 24 leads of the used grid electrode (Unique Medical Co., Ltd., Tokyo, Japan) covering both the frontal (premotor and motor cortex) and temporal (anterior two-thirds of the temporal lobe) convexity (Fig. 1C). Upon completion of ECoG recordings, anesthesia was changed back to TIVA, and for reduction of sevoflurane concentration, the flow rate of the respirator was set at 10 l/min without changes of FiO_2_. A temporary decrease of BIS value from 42–44 to 23–27 was noted within approximately 15 min after interruption of the sevoflurane inhalation and re-administration of propofol (Table 2). In 25 min after return to TIVA, MEP amplitude was about half of those at baseline, and 10 min later, it was restored fully in the upper extremity. Thereafter, anterior temporal lobectomy including lateral and medial temporal lobe structures was attained under appropriate MEP monitoring, which did not demonstrate changes of amplitude or latency (Fig. 1D). Upon completion of brain resection, propofol administration was stopped again, the end-tidal sevoflurane concentration was set at 2.5%, and 10 min later, control ECoG was recorded. Complete cessation of the previously observed epileptogenic activity from the frontal cortex was noted (Fig. 1E), which, obviously, indicated effective resection of the epileptic focus; therefore, additional frontal lobectomy was considered unnecessary.

**Table 1:** Dynamics of the anesthesia and monitoring parameters during switching from the total intravenous anesthesia to sevoflurane

Anesthesia parameters	Time from the initiation of switching from TIVA to sevoflurane (min)				
	0	2	5	10	15
Effect-site concentration of propofol (µg/ml)	2.30	2.03	1.65	1.22	1.03
End-tidal sevoflurane concentration (%)	0	2.1	2.2	2.5	2.5
Effect-site concentration of remifentanil (ng/ml)	7.28	7.25	6.89	6.49	6.32
BIS value	43	34	42	43	44
MEP amplitude (µV):					
- Left upper extremity (thenar muscles)	100	-	0	0	0
- Left lower extremity (anterior tibial muscle)	165	-	13	0	0
ECoG recording	-	-	Start	Recording	Finish

*BIS* Bispectral index, *ECoG* Electrocorticography, *MEP* Motor evoked potentials, *TIVA* Total intravenous anesthesia

**Table 2:** Dynamics of the anesthesia and monitoring parameters during switching from sevoflurane to the total intravenous anesthesia

Anesthesia parameters	Time from the initiation of switching from sevoflurane to TIVA (min)				
	5	10	15	26	37
Effect-site concentration of propofol (µg/ml)	3.01	2.24	2.26	2.30	2.30
End-tidal sevoflurane concentration (%)	0.14	0.13	0.11	0	0
Effect-site concentration of remifentanil (ng/ml)	6.23	6.18	6.15	6.13	6.12
BIS value	25	23	27	43	46
MEP amplitude (µV):					
- Left upper extremity (thenar muscles)	0	0	0	62	100
- Left lower extremity (anterior tibial muscle)	0	0	0	74	94

*BIS* Bispectral index, *MEP* Motor evoked potentials, *TIVA* Total intravenous anesthesia

Upon completion of the surgery, the patient demonstrated a flawless awakening from anesthesia and an uneventful postoperative period. No seizures were noted during subsequent medium-term follow-up.

## Discussion

In the presented case of resective epilepsy surgery, for effective and safe elimination of the epileptic focus without deterioration of the motor function, particularly caused by damage of the anterior choroidal artery, it was crucially important to attain both intraoperative ECoG recordings with induction of the epileptogenic activity and MEP monitoring. Therefore, a novel technique of intermittent switching TIVA and sevoflurane was applied.

Upon opening of the dura mater and interruption of the propofol administration, the baseline ECoG recordings were attained with the end-tidal sevoflurane concentration of 2.5% (corresponding to 1.5 minimum alveolar concentration [MAC]). It revealed induced synchronized high-amplitude epileptiform activity within the wide area of both the frontal and temporal cortex. Such an effect, which may resemble ECoG findings during an epileptic seizure, was specifically analyzed by Kurita et al. [5], who also demonstrated that a decrease of sevoflurane concentration diminishes the area of spikes’ occurrence and may better define their location at the onset of seizure. On the other hand, an increase of the end-tidal sevoflurane concentration up to 3.1–3.4% results in the rise of both the number of induced spikes and the frequency of their appearance [6]. It may be presumed that if a sufficiently large amount of propofol remains in the body, it will result in a more or less prominent suppression of epileptic spikes since their significant reduction was observed even after a single propofol dose of 2 mg/kg [7] and such anticonvulsant effects are seemingly mediated by GABA and NMDA receptors [8]. Although exact blood concentrations of propofol during surgery in our patient were not available, dynamic estimation of its effect-site concentrations using simulation software demonstrated a drop from 2.30 to 1.65 μg/ml within 5 min after interruption of administration and switching to sevoflurane, which allowed for informative ECoG recordings.

Nevertheless, sevoflurane is not suitable for effective MEP monitoring. It was shown that administration of this inhalational anesthetic at 0.3, 0.5, and 0.7 MAC results in corresponding decreases of the MEP amplitude to 66.2%, 41.3%, and 25.3% of the control [9]. It was clearly confirmed in our patient since administration of sevoflurane resulted in flattening of MEP. Such an effect was gradually reversed nearly to the baseline level within approximately 30 min after switching the anesthesia back to TIVA when the end-tidal sevoflurane concentration was 0%. It allowed for effective MEP monitoring during subsequent brain resection.

In full accordance with the contemporary practice of neuroanesthesia with TIVA, continuous infusion of remifentanil 0.25 μg/kg/min was attained in our patient during the entire procedure. In fact, the effects of opioids on induction of seizures may be comparable to those of sevoflurane and may be realized through inhibition of the GABAergic hippocampal neurons. McGuire et al. [8] analyzed ECoG during epilepsy surgery performed under general anesthesia attained by means of isoflurane and nitrous oxide with administration of droperidol and found that upon interruption of those anesthetics (with the end-tidal isoflurane concentration of zero), remifentanil at the highest plasma concentration of 4 ng/ml effects in noticeable increase of the spikes’ number. In our patient, according to the Minto pharmacokinetic model effect-site, concentrations of remifentanil during switching from TIVA to sevoflurane and vice versa were rather stable (range, 6.12–7.28 ng/ml).

There were concerns that switching from TIVA to inhalational anesthetic and vice versa during surgery may cause instability of circulatory dynamics and/or the depth of anesthesia. Therefore, during the entire procedure, the mean arterial pressure was monitored constantly and kept stable around the baseline level by means of the vasopressor administration. On the other hand, we observed a decrease of BIS value, which was most prominent during approximately 15 min after the change of sevoflurane to TIVA, while it returned to baseline values thereafter. Seemingly, such fluctuations of BIS values might be caused by temporary additive effects from the combination of anesthetic drugs [10]. Changes of the depth of anesthesia may have an important impact on postoperative recovery. Specifically, significantly higher incidence (28% vs. 19%) of postoperative delirium and worse cognitive function at 1-year follow-up were found in a subgroup of patients who were operated on under deep anesthesia (BIS value, 35) than under more superficial (BIS value, 50) one [11]. Nevertheless, despite the transient decrease of BIS values during surgery in our patient, he demonstrated an uneventful postoperative course and stable condition during subsequent follow-up.

Our technique presenting herein closely resembling those previously described by Fukamachi et al. [12], who used a similar intraoperative switch of TIVA to sevoflurane during callosotomy for intractable epilepsy in a pediatric patient. However, in difference with those authors, we have applied such a strategy for resective epilepsy surgery with an objective to localize precisely the epileptic focus and to guide its resection with the preservation of eloquent and non-affected cerebral cortex. To attain these goals, during surgery, we have switched anesthetic agents several times. Currently, we are collecting similar cases for their further detailed analysis. In any case, described intraoperative switch of anesthetic agents according to specific intraoperative requirements seems rather useful for cases of brain surgery requiring both ECoG recordings and MEP monitoring.

## Data Availability

Reported data may be shared upon reasonable request of the medical professionals addressed to the corresponding author.

## Abbreviations

BIS: Bispectral index
ECoG: Electrocorticography
EEG: Electroencephalography
MAC: Minimum alveolar concentration
MEP: Motor evoked potentials
SEEG: Stereoelectroencephalography
TCI: Target-controlled infusion
TIVA: Total intravenous anesthesia
